# Oversight of Pathogen Research Must Be Carefully Calibrated and Clearly Defined

**DOI:** 10.1128/jvi.00176-23

**Published:** 2023-02-27

**Authors:** Anice C. Lowen, Arturo Casadevall, James C. Alwine, Lynn W. Enquist, Felicia D. Goodrum, Michael J. Imperiale, Seema S. Lakdawala

**Affiliations:** a Department of Microbiology and Immunology, Emory University School of Medicine, Baltimore, Maryland, USA; b Department of Microbiology and Immunology, Bloomberg School of Public Health, Johns Hopkins University, Baltimore, Maryland, USA; c Department of Cancer Biology, University of Pennsylvania School of Medicine, Philadelphia, Pennsylvania, USA; d Department of Immunobiology, University of Arizona, Tucson, Arizona, USA; e Department of Molecular Biology, Princeton University, Princeton, New Jersey, USA; f Department of Microbiology and Immunology, University of Michigan, Ann Arbor, Michigan, USA

**Keywords:** biosecurity, microbiology, oversight, pathogen, virology

## EDITORIAL

By providing consultation to the United States Government (USG) on matters of biosecurity, the National Science Advisory Board for Biosecurity (NSABB) plays a critical role in ensuring the safety of biomedical research in the United States.

In February 2022, the NSABB was tasked with evaluating and providing recommendations on two current frameworks for USG oversight of research on pathogens of high consequence: Dual Use Research of Concern (DURC) (1) and Potential Pandemic Pathogen Care and Oversight (P3CO) (2). In response to this USG request, on 27 January 2023, the Board voted to approve 13 recommendations with minor modifications (3). If adopted, these recommendations will vastly change federal oversight of USG-funded research on pathogens.

Here, we restate the recommendations of the Board and give our response to each, with the goals of promoting modification of some aspects and of providing a resource to USG entities charged with implementing new policies that follow from the recommendations. We provide these responses out of concern for the potential consequences of excessive oversight: policy that unduly restricts microbiology regardless of associated risk will limit our preparedness for and ability to respond to public health threats, constrain efforts to control infectious diseases of all kinds, diminish our international engagement in these critical global issues, and damage U.S. economic competitiveness.

## RESPONSES TO RECOMMENDATIONS

*Recommendations 1–7 relate to the Board’s review of the current P3CO framework.*

**Recommendation 1.** Amend USG P3CO policy to clarify that federal department-level review is required for research that is reasonably anticipated to enhance the transmissibility and/or virulence of any pathogen (i.e., PPPs and non-PPPs) such that the resulting pathogen is reasonably anticipated to exhibit the following characteristics that meet the definition of a PPP:

• Likely moderately or highly transmissible and likely capable of wide and uncontrollable spread in human populations; and/or
• Likely moderately or highly virulent and likely to cause significant morbidity and/or mortality in humans;

And, in addition

• Likely to pose a severe threat to public health, the capacity of public health systems to function, or national security.

RESPONSE: *The recommendation to expand P3CO oversight to all pathogens proposes a massive increase in the scope of federal oversight of microbiology. While this is clearly the Board’s intention*, *we are deeply concerned that the logistical challenges of implementing such an expansion make widespread disruption to affected fields of study unavoidable.*

*First*, *we caution that*, *unless the types of experiments that do and do not require review are clearly defined*, *abundant unnecessary review will obstruct important research*, *leaving the United States unprepared for infectious disease threats due to the much-slowed pace of virology research*, *and perhaps all of microbiology. To give one example*, *some pathogens lose virulence under laboratory conditions and must be passaged in animals to maintain virulence. Based on the NSABB’s recommendation*, *this routine practice may now fall into the USG P3CO policy*, *adding heavy administrative oversight to some fields. We note that “virulence” is a relative term inasmuch as it is always measured relative to another strain or pathogen. Imprecise terminology can cause confusion and thus increase administrative burden unnecessarily.*

*Second*, *the term “reasonably anticipated” is vague and subjective. It will be even more problematic if*, *as proposed*, *additional responsibilities for review are imposed on institutions. At present*, *the DURC guidelines give institutions a discrete list of agents for which additional review is warranted. In the absence of such clear-cut categories*, *there will be a lack of consistency among institutions in how oversight is applied. As a consequence*, *routine*, *low-risk experimental approaches will be delayed. Very specific guidance and definitions must be developed.*

*Third*, *there is a fundamental problem with the idea that the experimental outcomes referred to can be reasonably anticipated. Correlates of transmission and virulence are incompletely understood even for the best-studied pathogens. In addition*, *most available information was collected in model systems that*, *while useful in many circumstances*, *present limitations for translation to natural hosts and for the prediction of pathogen dynamics at a population level. It is thus unclear how even a well-informed oversight committee will meaningfully evaluate the potential for research to yield an agent that meets any of the three criteria outlined.*

*Fourth*, *if this recommendation is adopted*, *its implementation must be carefully designed to avoid entrapping research in a labyrinth of confusing and drawn-out bureaucratic processes. We suggest the following guiding principles for implementation:*

(i) *Oversight at a local level will be most efficient. We note that much of this research is already reviewed at the local level. Instead of mandating federal review, institutional biosafety committees (IBCs)* *could be educated on how to interpret federal guidance and tasked with implementing it. Compliance could be assured at the IBC level.*
(ii) *Federal oversight should be scaled according to the level of risk identified by investigators and IBCs. Research determined not to fall into one of the seven categories described in Section IV.C of the current P3CO framework should move forward without the need for federal approval.*
(iii) *Investigators and institutions should be provided with clear guidance on the interpretation of the seven P3CO categories. This guidance should be developed in consultation with subject matter experts and then piloted to identify areas of confusion or ambiguity before being rolled out comprehensively. This is essential to avoid wide inconsistency in decision-making across institutions.*

**Recommendation 2.** Remove current blanket exclusions for research activities associated with surveillance and vaccine development or production. However, include and implement processes and procedures for urgent federal department level review and evaluation of ePPP research critical for public health or national security.

RESPONSE: *This is a problematic recommendation because surveillance and vaccine research constitute the major pillars of society’s defenses against PPPs, and there is concern that removing the exclusions will make this research even more difficult. Could the rapid progress in both SARS-CoV-2 surveillance and vaccine development that we witnessed in the first year of the COVID-19 pandemic have been possible if this oversight framework had been in place? Even if additional review is expedited*, *it will impede timely surveillance and characterization of viruses emerging*, *variants arising*, *and vaccine development. These activities are critical responses to infectious-disease outbreaks and offer little benefit to public health if carried out retrospectively. Surveillance must be able to keep pace with pathogen spread. Vaccine development should ideally outpace pathogen spread and be rapid enough to allow updating of vaccines in response to pathogen evolution. We urge careful consideration of the impact of this recommendation on the annual updating of seasonal influenza vaccines and the newly proposed similar process for SARS-CoV-2 vaccines*, *especially in light of the broad*, *international surveillance network that supports this important effort. At a minimum*, *the specific surveillance and vaccine research activities that would need additional oversight should be well defined for both institutions and investigators.*

**Recommendation 3.** **(i) Recommendation 3.1.** Amend the USG P3CO framework to include and articulate specific roles, responsibilities, and expectations for investigators and institutions in the identification, review, and evaluation of research for potential involvement of ePPPs, taking into account existing review and oversight processes.

RESPONSE: *We agree with the Board that the paucity of information on specific roles in the current P3CO framework is problematic and that the clear expectations articulated in the DURC framework offer a good example to follow. As noted above*, *we encourage concentration of oversight at the local IBC level with defined guidelines for standardization across institutions.*

**(ii) Recommendation 3.2.** Local, institutional compliance procedures must be better harmonized, strengthened where needed, and adequate technical and financial assistance provided.

RESPONSE: *We agree. Inconsistent interpretation of guidance across institutions needs to be avoided through the provision of clearly defined*, *objective guidelines*, *pertinent examples*, *training*, *and funding to allow expansion of biosafety expertise at research institutions.*

**(iii) Recommendation 3.3.** Designate a USG office with adequate technical and financial support to assist investigators and institutions in the review process to ensure consistent evaluation of PPP status.

RESPONSE: *Again*, *we urge concentration of oversight processes at the institutional level but agree that USG partners should be well resourced to avoid creating a bottleneck in the review process.*

**Recommendation 4.** **(i) Recommendation 4.1.** Amend the OSTP P3CO Policy Guidance to be consistent with the Belmont Report (4) and amend the HHS P3CO Framework to clarify that the seven categories of research outlined must be given extra care and considered throughout the life of the research, including the proposal, review, evaluation, and ongoing oversight process. In addition, develop principles and guidelines that can be applied and implemented to ensure, 1) there are no feasible alternative methods of obtaining the relevant benefits from proposed research that poses less risk; and 2) unnecessary risks have been eliminated and the remaining risks are justified by the potential benefits.

RESPONSE: *While we agree that ethical principles need to be followed in all walks of life*, *the analogy to the Belmont Report here is ill-advised. The oversight of human subject research is a very different matter from the oversight of basic laboratory research. Furthermore*, *there is consensus in the medical establishment that the Institutional Review Board (IRB) system has created a bureaucracy that needs reform* (5). *The IRB network for oversight of human research may be contributing to the slowing pace of medical innovation* (6).

*Often*, *it is not possible to eliminate all risks. We suggest modification of this recommendation to state, “Risks that are not necessary to answer an important scientific question have been appropriately considered and mitigated… .”*.

**(ii) Recommendation 4.2.** Develop an implementation directive/plan, additional guidance, educational materials, and standard operating procedures, including ongoing review, evaluation, and oversight procedures and criteria that can be used or adapted by funding institutions, research institutions, and investigators when implementing the policy.

RESPONSE: *We agree that the broad oversight proposed will require the development of extensive guidance and training materials and that the Government entity charged with implementation should seek to facilitate the adoption of the new processes by providing well-thought-out tools and resources to institutions and investigators. In addition*, *we caution that putting excessive burdens on institutions in the oversight of research on pathogens may cause some less well resourced institutions to discontinue research in microbiology*, *at great cost to the scientific enterprise and to the detriment of humanity’s ability to combat infectious disease threats. To mitigate this risk*, *we suggest funneling financial resources to institutions to allow them to build and maintain the necessary oversight structures as well as relying on subject matter experts to help inform and interpret the risks and benefits of proposed research.*

**Recommendation 5.** Take additional steps to increase transparency in the review process at the federal and local levels, including sharing a summary of key determinants that informed ePPP research funding decisions.

RESPONSE: *IBCs already include members of the community (i.e., individuals who have no affiliation with or connection to the institution), and we agree that this is an important practice. We caution that transparency needs to be balanced with the need to protect investigators from attack by individuals with anti-science agendas and those who may view research on pathogens as conspiracy.*

**Recommendation 6.** Consider development of analogous policies and processes for identification, review, evaluation, and ongoing oversight of relevant research involving enhanced pathogens likely to pose severe threats to human health, food security, economic security, or national security by its impacts on animals or plants or to animal or plant products.

RESPONSE: *We agree that pathogens of nonhuman animals and plants are of primary importance to humanity and global ecosystems and should not be overlooked in the development of disaster prevention and preparedness strategies. However*, *we again raise concern over the scale of the proposed expansion of oversight. Unless the types of experiments that do and do not require review are clearly defined*, *abundant unnecessary review will obstruct critical research. In defining this scope*, *it will be important to define the term “pathogen” in light of the ubiquity of microbes in living systems; this is another area of dangerous ambiguity in the draft recommendations.*

**Recommendation 7.** The conduct of ePPP research at international institutions receiving USG support for life sciences research, either directly or indirectly (e.g., via subawards or contracts), must also be subject to review, evaluation, and ongoing oversight procedures that are equivalent to domestic U.S. policies and procedures.

RESPONSE: *We agree.*

*Recommendations 8–13 relate to the Board’s review of the current DURC framework.*

**Recommendation 8.** **(i) Recommendation 8.1.** Continue to facilitate sharing of experiences and best practices regarding DURC policy implementation.

RESPONSE: *We agree.*

**(ii) Recommendation 8.2.** Any updates to USG DURC policies, particularly updates regarding the scope of research subject to review and/or the relevant entities to which the policies apply, must involve relevant stakeholders and be accompanied by robust USG outreach and education and an adequate implementation period.

RESPONSE: *We agree.*

**Recommendation 9.** Remove the term “directly misapplied” from the DURC definition, which may not be beneficial to, and could potentially limit the identification and oversight of research that may pose significant threats, whether deliberate or accidental in nature.

RESPONSE: *We agree.*

**Recommendation 10.** **(i) Recommendation 10.1.** Expand the scope of research requiring review for potential DURC to include research that directly involves any human, animal, or plant pathogen, toxin, or agent that is reasonably anticipated to result in one or more of the seven experimental effects.

RESPONSE: *We do not disagree in principle with uniform oversight across all pathogens. Nevertheless*, *we are very concerned about the feasibility of implementing this practice without major disruption to research. We urge clear and focused definition of the types of research that require review. We note the use of the ambiguous terms “reasonably anticipated” and “pathogen” again in this recommendation.*

**(ii) Recommendation 10.2.** Establish mechanisms and processes to ensure that investigators and institutions are executing their responsibilities effectively.

RESPONSE: *We agree and recommend that*, *to be successful*, *these mechanisms take the form of constructive partnership.*

**(iii) Recommendation 10.3.** Review of bioinformatics, modeling, and other *in silico* experimental approaches and research involving genes from or encoding pathogens, toxins, or other agents for potential DURC is not recommended at this time. However, investigators and institutions should be aware of the potential risks of such research and continued assessment of the risks and benefits associated with advances and applications of such approaches must inform the ongoing evaluation of the scope of these policies.

RESPONSE: *We agree at this time.*

**Recommendation 11.** Engage relevant stakeholder and publishing groups to encourage development and adoption of more uniform editorial policies, review processes, and best practices for identifying material that may raise significant biosecurity and biosafety concerns and facilitate the sharing of best practices and guidelines for assessing options for mitigating risks.

RESPONSE: *We agree with this recommendation in principle. Some publishing organizations like the ASM already have protocols in place for the review of DURC in their journals* (7). *However*, *as much of the scientific publishing industry is overseas*, *this would require international cooperation. Furthermore*, *as we learned from the H5N1 transmissibility studies*, *the publication step is too late in the research life cycle to prevent potential misuse of information. In the absence of uniform standards for all journals*, *if a manuscript is rejected because of DURC concerns*, *authors will publish elsewhere. While DURC concerns should be addressed at every stage of the research life cycle*, *oversight must be concentrated on the front end.*

**Recommendation 12.** In line with the NSABB’s 2016 recommendation regarding ePPP research, promote and ensure that all research meeting the scope of these policy frameworks conducted within the U.S. and/or supported by the U.S. government be subject to equivalent oversight regardless of funding source.

RESPONSE: *We agree.*

**Recommendation 13.** Develop an integrated approach to oversight of research that raises significant biosafety and biosecurity concerns, including ePPP research and DURC. Clearly articulate federal, institutional, and investigator responsibilities in the assessment and identification of proposed and ongoing research, and minimize the potential for duplicative or parallel institutional or federal review processes.

RESPONSE: *We agree. In particular*, *we agree that there is currently duplication of effort in DURC and P3CO review and welcome integration of these frameworks into a single oversight mechanism.*

## SUMMARY

While a number of the Board’s recommendations are well placed, the recommended extension of P3CO and DURC to all pathogens, the removal of exclusions for surveillance and vaccine research, and the ambiguous definition of experiments requiring oversight combine to engender great concern. Unnecessary review would slow most microbiology research in the United States, needlessly increase its cost and administrative burden, and make microbiology unattractive for upcoming scientists who might have entered the field. Thus, many of the greatest microbiology research enterprises in the world will be impaired. Recall the massive human and economic tolls caused each year by microbial diseases of humans, animals, and plants. A hobbled U.S. microbiology enterprise would be unable to do research that might reduce these tolls. While microbiology research waits in line in the U.S., other countries would move forward, and the U.S. would become beholden to others for pandemic detection and preparedness, vaccine development and supply, antimicrobial drug development, and basic microbiology knowledge. The economic and national security costs and consequences of this scenario would be great, all resulting from ill-defined oversight. It is essential that pathogen research be safe, but the oversight to ensure safety must be balanced and rely on an evidence-based framework that maintains and expands much-needed microbiology research in the United States.
